# Magnetic Resonance Enterography and Intestinal Ultrasound for the Assessment and Monitoring of Crohn’s Disease

**DOI:** 10.1093/ecco-jcc/jjae042

**Published:** 2024-03-30

**Authors:** Shankar Kumar, Isabelle De Kock, William Blad, Richard Hare, Richard Pollok, Stuart A Taylor

**Affiliations:** Centre for Medical Imaging, University College London, London, UK; Radiology Department, Ghent University Hospital, Ghent, Belgium; Department of Gastroenterology, University College Hospitals NHS Foundation Trust, London, UK; Faculty of Biology, Medicine and Health, University of Manchester, Manchester, UK; Department of Gastroenterology, St George’s University Hospitals NHS Foundation Trust, London, UK; Centre for Medical Imaging, University College London, London, UK

**Keywords:** Inflammatory bowel disease, Crohn’s disease, magnetic resonance enterography, intestinal ultrasound

## Abstract

Magnetic resonance enterography [MRE] and intestinal ultrasound [IUS] have developed rapidly in the past few decades, emerging as the primary non-invasive options for both diagnosing and monitoring Crohn’s disease [CD]. In this review, we evaluate the pertinent data relating to the use of MRE and IUS in CD. We summarise the key imaging features of CD activity, highlight their increasing role in both the clinical and the research settings, and discuss how these modalities fit within the diagnostic pathway. We discuss how they can be used to assess disease activity and treatment responsiveness, including the emergence of activity scores for standardised reporting. Additionally, we address areas of controversy such as the use of contrast agents, the role of diffusion-weighted imaging, and point-of-care ultrasound. We also highlight exciting new developments, including the applications of artificial intelligence. Finally, we provide suggestions for future research priorities.

## 1. Introduction

The management of Crohn’s disease [CD] utilises a ‘treat-to-target’ strategy, with therapy modified according to objective measures of disease activity that are assessed at regular time intervals.^1–4^ Treatment is targeted at achieving both biochemical and endoscopic remission.^5^ Whereas endoscopy is the primary method for evaluating disease activity, it has several limitations that preclude its repeated use. It is invasive with the potential for severe complications, has low patient tolerability, and can be technically very difficult to perform in the presence of strictures, adhesions, or severe inflammation.^6,7^ Additionally, endoscopy can be falsely negative in cases of proximal small bowel disease, and it does not assess extra-intestinal disease, which is present in nearly half of patients with inflammatory bowel disease [IBD].^8,9^ As a result, magnetic resonance enterography [MRE] and intestinal ultrasound [IUS] have developed rapidly in the past few decades, emerging as the primary non-invasive options for both diagnosing and monitoring CD, particularly since they mitigate against the cumulative risk of exposure to diagnostic medical radiation in the management of this long-term condition.^10–13^ Both modalities have high sensitivity for detecting active CD, and are endorsed by multiple international guidelines as appropriate first-line investigations and viable alternatives to colonoscopy.^11,14,15^ Indeed, transmural healing is an increasingly important endpoint in clinical trials.^3,16^

Magnetic resonance imaging [MRI] of the small bowel offers a high-tissue–contrast examination of the abdomen and pelvis with multiplanar assessment, without exposure to diagnostic medical radiation, which is a disadvantage of CT.^10^ Furthermore, where necessary, it can simultaneously evaluate perianal complications.^17–19^ MRE provides high diagnostic accuracy for detecting the presence and activity of CD, with reasonable inter-observer agreement between radiologists.^11,20–24^ IUS also benefits from not conferring exposure to diagnostic medical radiation, as well as being favoured by patients because it is quick to perform and does not usually require any bowel preparation. It too is highly sensitive and specific for identifying the presence of CD and evaluating disease activity. Typical protocols for MRE and IUS are provided in Supplementary Table 1.

In this article, we review and evaluate the key data related to the use of MRE and IUS in the diagnosis and management of CD. We describe the typical imaging features of CD activity and emphasise the increasing use of these imaging techniques in both clinical and research settings. We discuss how these modalities fit within diagnostic pathways, offering guidance about test choice. We consider how to use them in assessing treatment response, and address the emergence of disease activity scoring systems which aim to standardise evaluation and therapeutic response in CD. Finally, we address areas of controversy and draw attention to promising new areas of research, with some suggestions for future research priorities.

## 2. Cross-sectional Imaging Signs of Disease Activity

The diagnostic features of CD on cross-sectional imaging are well described and depend on factors such as inflammatory burden, existing bowel damage, and the presence of complications such as stricturing or penetrating disease.^25^ In mild disease, cross-sectional imaging may not reveal any abnormality, particularly as superficial aphthous ulceration is often not apparent. Consensus guidelines recommend using specific nomenclature when interpreting cross-sectional imaging, to improve reporting consistency.^26–28^ One of the major advances made possible by the widespread use of cross-sectional imaging in IBD is the ability to measure transmural disease activity. By examining the full thickness of the bowel wall and surrounding tissues, imaging can detect features that are not visible when the evaluation is limited to the mucosa alone.^29^ Multiple radiological features of active CD have been validated against endoscopy, histopathology, and inflammatory markers in blood and stool.^30^ Such signs are employed during routine clinical reporting, but also form the basis of disease activity scores [see below].

**Figure 1:**
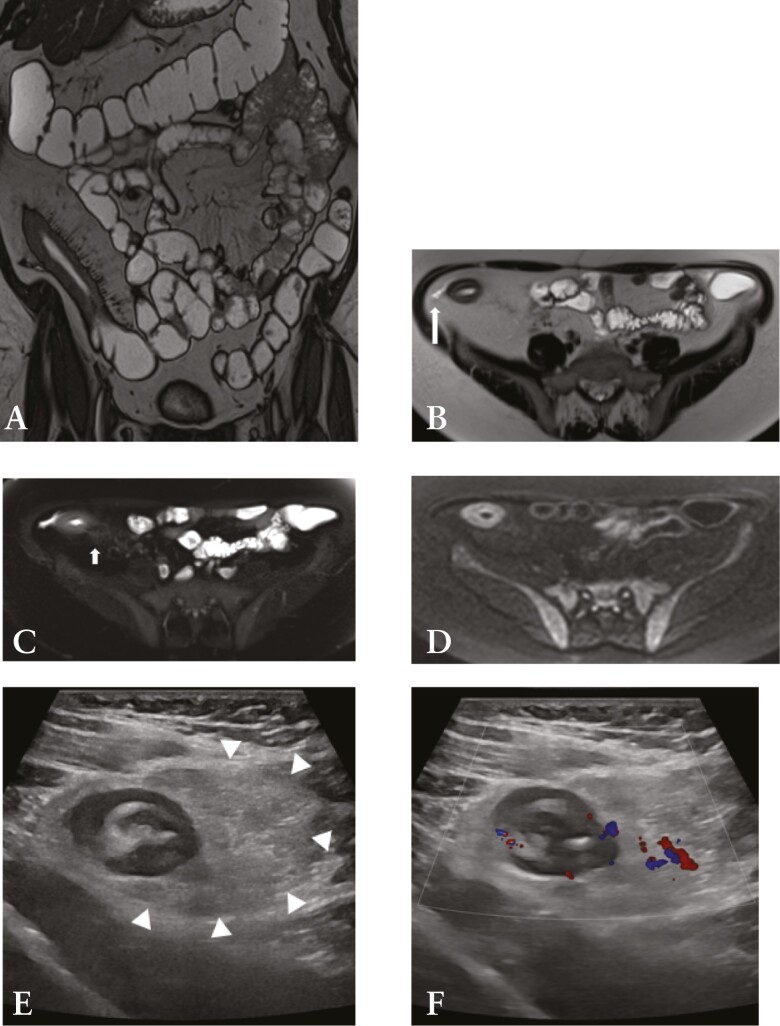
MRE [A–D] and IUS [E–F] images in a 56-year-old female with Crohn’s disease. A: Coronal T2 TRUFI image demonstrating bowel wall thickening at the terminal ileum and engorged vasa recta. B: Axial T2 HASTE image showing mural oedema in the terminal ileum, perienteric fluid [long arrow], and fat wrapping. C: Axial T2 HASTE image with fat saturation facilitates detection of the presence of both mural oedema and mesenteric oedema [short arrow]. D: Axial DWI [b600] image showing restricted diffusion in the inflamed terminal ileum. E: Greyscale IUS image showing bowel wall thickening at the terminal ileum with loss of mural stratification and presence of fat wrapping [arrowheads]. F: Colour Doppler image demonstrating hyperaemia in the thickened terminal ileal wall extending into the hypertrophic mesenteric fat. MRE, magnetic resonance enterography; IUS, intestinal ultrasound; DWI, diffusion-weighted imaging.

Bowel wall thickening is an important and early finding in active CD inflammation, observed on both MRE and IUS. A recent consensus panel concluded that bowel thickening is present when the bowel wall is thicker than 3 mm. However, this finding is non-specific and can be caused by various pathological processes affecting the gut, including infectious and neoplastic aetiologies.^31^ In CD, it results from inflammatory cell infiltrate or bowel wall oedema, with or without the presence of fibrosis, and is likely the most sensitive marker of inflammatory activity.^26^ Notwithstanding, given the nearly universal concurrence of inflammatory and fibrotic changes in CD, other more sensitive parameters for active CD must also be taken into account. On MRE, neo-angiogenesis and increased vascularisation are represented by increased mural enhancement following intravenous gadolinium injection, as well as engorgement of the vasa recta, and on IUS, increased colour Doppler signal is observed [Figure 1]. Mural and transmural oedema can also be present in active CD. The former manifests on MRE as hyperintense T2 signal in the bowel wall, which is typically submucosal, and as disrupted mural stratification on IUS. Transmural oedema is reflected in both modalities by the presence of free fluid and perienteric fat abnormality.^25^ Fibro-fatty proliferation or fat wrapping refers to hypertrophy and expansion of the mesenteric fat towards the anti-mesenteric side, which produces a mass effect on the nearby bowel loops and is often seen in longstanding CD.^26^ Selective saturation of fat signal on T2-weighted sequences aids the identification of intestinal wall oedema and perienteric fat on MRE. Indeed, fat-saturated and non-fat-saturated T2 sequences are imperative to determine whether the increased mural signal intensity is due to the presence of oedema or intramural fat deposition, a phenomenon that occurs in longstanding CD. The former demonstrates high signal intensity on both sequences, whereas the wall signal intensity will reduce on the fat-saturated sequence in the context of fat infiltration [Figure 1]. On IUS, increased fat echogenicity is a sign of active CD. Ulceration can be detected on MRE if adequate luminal distension is achieved, seen as thin high signal intensity lines within the thickened bowel wall.^28^ On IUS, ulceration manifests as defects in the mucosal layer.

## 3. Disease Activity Scores

In an attempt to standardise imaging criteria and reduce reader subjectivity, to report and quantify active CD, a variety of MRE and IUS activity scores have been developed [Table 1] and validated [Table 2].^30^ These indices are comprised of similar individual components, with substantial interrater reliability reported.^38^ Scoring systems like these are an attractive proposition as they provide a more objective and systematic assessment of the imaging findings, similar to endoscopic activity scores They hold considerable promise for use in therapeutic clinical trials.^34,39^ Currently, their use is mainly limited to a research/clinical trial setting.^40^ However, with their increasing simplicity, their wider use in clinical practice is likely to increase.

**Table 1:** Selected MRE and IUS activity scores.

Score	Formula	Variables				
sMARIA	[1 × wall thickness >3 mm] + [1 × wall oedema] + [1 × fat stranding] + [2 × ulcers]	Wall thickness >3 mm scores 1 point Presence of oedema scores 1 point Presence of fat stranding scores 1 point Presence of ulcers scores 2 points				
London	1.79 + [1.34 × mural thickness] + [0.94 × mural T2 score]					
‘Extended’ London	Mural thickness + mural T2 score + perimural T2 signal + contrast enhancement		0	1	2	3
		Mural thickness	1–3 mm	>3–5 mm	>5–7 mm	>7 mm
		Mural T2 score	Equivalent to normal bowel wall	Minor increase in signal: bowel wall appears dark grey on fat-saturated images	Moderate increase in Signal: bowel wall appears light grey on fat-saturated images	Marked increase in Signal: bowel wall contains areas of white high signal approaching that of luminal content
		Perimural T2 signal	Equivalent to normal mesentery	Increase in mesenteric signal but no fluid	Small fluid rim [≤2mm]	Larger fluid rim [>2mm]
		Enhancement	Equivalent to normal bowel wall	Minor enhancement—bowel wall signal greater than normal small bowel but significantly less than nearby vascular structures	Moderate enhancement—bowel wall signal increased but somewhat less than nearby vascular structures	Marked enhancement—bowel wall signal approaches that of nearby vascular structures
BUSS	0.75 × bowel wall thickness + 1.65 × bowel wall flow	Bowel wall thickness in mm Bowel wall flow—defined as [0] absence or [1] presence of vascular signals at colour Doppler				
SUS-CD	Bowel wall thickness + colour Doppler score		0	1	2	3
		Bowel wall thickness	<3 mm	3–4.9 mm	5–-7.9 mm	≥8 mm
		Colour Doppler score	No or single vessel per cm^2^	2–5 vessels per cm^2^	>5 vessels per cm^2^	NA
IBUS-SAS	4 × bowel wall thickness + 15 × inflammatory fat + 7 × colour Doppler score + 4 × bowel wall stratification	Bowel wall thickness in mm				
			0	1	2	3
		Inflammatory fat	Absent	Uncertain	Present	NA
		Colour Doppler score	Absent	Short signal	Long signals inside bowel	Long signals inside and outside bowel
		Bowel wall stratification	Normal	Uncertain	Focal [≤3 cm]	Extensive [>3 cm]

MRE, magnetic resonance enterography; IUS, intestinal ultrasound; NA, not applicable; BUSS, bowel ultrasound score; sMARIA, simplified magnetic resonance index of activity; SUS-CD, simple ultrasound score for Crohn’s disease; IBUS-SAS, International Bowel Ultrasound Segmental Activity Score.

**Table 2:** Prospective external validation of selected MRE and IUS scoring systems.

Study	Reference standard	Sample size/number of centres/number of readers	Scoring system evaluated	Treatment/time of repeat evaluation	Main results	Strengths	Limitations
Puylaert *et al*., 2019^32^	eAIS/CDEIS	98 patients, all TI segments/2/2	London	NA	Sensitivity for active disease = 79%/82% Specificity for active disease = 63%/71%	Two robust reference standards	Only TI assessed Expert readers No assessment of responsiveness
Capozzi *et al*., 2020^33^	CDEIS	50 patients, 42 TI segments, 228 colonic segments at baseline, 39 patients at time of reassessment/1/2	sMARIA	TNF inhibitors, vedolizumab, ustekinumab/46 weeks	Sensitivity at baseline for active/severe disease/treatment responsiveness = 88.9%/86.9%/89.5% Specificity at baseline for active/severe disease/treatment responsiveness = 93.4%/91.9%/87.5%	Robust reference standard Assessment at baseline and following treatment	No patients with mild CD Single centre
Hanzel *et al*., 2022^34^	CDEIS	41 patients, 18 TI segments, 11 colonic segments, 12 ilecolonic segments/1/3	sMARIA	Adalimumab, infliximab, vedolizumab, corticosteroids/12-14 weeks	SES for sMARIA = 1.17 [95% CI 0.56 to 1.77], London = 0.85 [95% CI 0.31 to 1.39], ‘extended’ London = 0.95 [95% CI 0.38 to 1.51]	Robust reference standard Assessment at baseline and following treatment	Single centre Different drugs Expert readers
Kumar *et al*., 2022^35^	HAI	111 patients, all TI segments/7/26	sMARIA, London, ’extended’ London	NA	Sensitivity for active disease sMARIA/London/‘extended’ London = 83%/76%/81% Specificity for active disease sMARIA/London/‘extended’ London = 41%/64%/41% Sensitivity/specificity of sMARIA for severe disease = 84%/53%	Numerous centres and readers Not highly specalised readers	Histological reference standard No assessment of responsiveness
Dragoni *et al*., 2023^36^	SES-CD, Rutgeerts score in case of bowel resection	73 patients, 21 ileal segments, 5 colonic segments, 47 ileocolonic segments/1/1	IBUS-SAS, BUSS, SUS-CD	NA	Sensitivity for active disease IBUS-SAS/BUSS/SUS-CD = 82.2%/91.1%/93.3% Specificity for active disease IBUS-SAS/BUSS/SUS-CD = 100%/82.1%/71.4%	Robust reference standard	Single expert reader No assessment of responsiveness
Kumar et al., 2024^37^	HAI/sMARIA	111 patients for the histology reference, 284 for the MRE reference standard, all TI segments/8/19	SUS-CD, BUSS	NA	Against histology, sensitivity/specificity: SUS-CD = 79%/50% BUSS = 66%/68% Against MRE, sensitivity/specificity: SUS-CD = 81%/75% BUSS = 68%/85%	Numerous centres and readers Not highly specalised readers Two reference standards	No assessment of responsiveness

BUSS, Bowel Ultrasound Score; CD, Crohn’s disease; CDEIS, Crohn’s Disease Endoscopic Index of Severity; eAIS, endoscopic Activity Index Score; IBUS-SAS, International Bowel Ultrasound Segmental Activity Score; NA, not applicable; SES-CD, Simple Endoscopic Score for Crohn’s Disease; sMARIA, Simplified Magnetic Resonance Index of Activity; SUS-CD, Simple Ultrasound Score for Crohn’s Disease; TI, terminal ileum; TNF, tumour necrosis factor.

### 3.1. MRE

The Magnetic Resonance Index of Activity [MARIA] encompasses wall thickening, mural contrast enhancement, mural oedema, and ulceration, all independent predictors of the presence and severity of endoscopic lesions.^10,41^ Cut-off values have been defined for both active disease [**≥7**] and severe disease] [**≥**11]. Limitations of the MARIA score include its time-consuming nature with the need to place regions of interest in the bowel wall, and the inclusion of normal bowel wall segments when calculating a global score, rendering the MARIA unwieldy for routine clinical practice.^42^ Such limitations led to the development of the more time-efficient simplified MARIA [sMARIA]; the time required to derive it is just 4.5 min compared with over 12 min for the MARIA.^43^ The sMARIA was derived and validated by Ordas *et al*. in a single-centre study comprising 98 patients, employing the CD endoscopic index of severity [CDEIS] as the reference standard.^44^ Sensitivity and specificity for identifying active disease were 90% and 81%, and 85% and 92% for severe disease, respectively. In patients who received anti-tumour necrosis factor agents or corticosteroids for 12 weeks, the sMARIA accurately identified endoscopic remission [CDEIS <3.5] with both sensitivity and specificity exceeding 90%.

Steward *et al*. derived and validated the London and ‘extended’ London scores against a histological standard of reference, the endoscopic biopsy acute inflammatory score [eAIS].^45^ This was a single-centre study comprising a total of 42 patients. The London score had a sensitivity of 81% [95% confidence intervals 54 to 96] and specificity of 70% [35 to 93] for detecting active terminal ileal CD, whereas the sensitivity and specificity of the ‘extended’ London scores were 87% [61 to 98] and 70% [35 to 93], respectively.

The sMARIA, London, and ‘extended’ London scores have similar parameters. However, the ‘extended’ London score requires gadolinium contrast, which is a limitation. The three activity scores have since been studied in both retrospective^42,46–48^ and prospective settings, with the latter external validation studies summarised in Table 2.^32–35,49^

Another MRE index is the Clermont score, which represents a reliable and accurate tool for assessing CD activity.^50^ There is much overlap between its constituents and the MARIA; the distinguishing feature of the Clermont score is its use of diffusion-weighted sequences [see below] rather than post-gadolinium imaging. The necessity to place a region of interest for its derivation, which is time-consuming, is likely to hinder its uptake in routine clinical practice, but it provides another option for clinical trials.^51^

As outlined, there are a range of MRE indices available, but there remains significant variation in how these are used and what is considered to represent treatment response and remission of CD by MRE.^52^ Consensus guidelines are needed to define such criteria for even more objective assessment in clinical trials.

### 3.2. IUS

In the same manner as MRE, a variety of IUS activity scores that include the most useful parameters have been developed, to make the assessment more systematic and reproducible.^53^ Most of these scores focus on bowel wall thickness, increased colour Doppler signal, disrupted mural stratification, and fat wrapping.^54,55^ The most promising IUS indices, namely the bowel ultrasound score [BUSS], the simple ultrasound score for Crohn’s disease [SUS-CD], and the International Bowel Ultrasound Segmental Activity Score [IBUS-SAS], are summarised in Table 1. Presently, these scores have undergone less prospective external validation than there MRE counterparts, although this is being increasingly addressed [Table 2].

The BUSS, comprising bowel thickness and colour Doppler signal, was developed in a cohort of 225 patients originating from a single centre.^56^ IUS was performed by one of two gastroenterologists who had at least 7 years of experience of US. The BUSS had a sensitivity of 83% (95% confidence intervals [CI] 76 to 88) and specificity of 85% [73 to 93] for the assessment of disease activity when compared with the reference standard of the simple endoscopic score for CD [SES-CD]. In a subsequent publication, the same authors demonstrated that the BUSS also performs well in assessing treatment responsiveness.^57^ They again employed the reference standard of SES-CD, and evaluated 48 CD patients from the same single centre who were starting a new therapy with a biologic or immunosuppressant. IUS was carried out by one of two gastroenterologists with at least 8 years of experience. Reassessment with IUS was undertaken at a median time of 13.3 months from baseline. Applying a cut-off value for <3.52 of BUSS for inactive disease, the sensitivity and specificity for identifying endoscopic remission following treatment were 90% [55 to 99] and 74% [58 to 87], respectively. Moreover, the BUSS changed significantly from baseline to follow-up in those patients achieving an endoscopic response. Indeed, a change of -1.2 in the BUSS from baseline to reassessment predicted endoscopic response with a sensitivity and specificity of 74% [49 to 91] and 83% [65 to 94], respectively.

The SUS-CD was developed in a single-centre study comprising 40 patients, using the SES-CD as the reference standard.^58^ As part of the same publication, the authors also performed validation via 124 patients from two other institutions. The same reference standard was employed, and two sonographers performed IUS. They reported sensitivity and specificity of 95.3% [88 to 98] and 70.3% [56 to 82], respectively.

The IBUS-SAS was developed by 11 international experts through a Delphi Consensus, followed by a blinded agreement study with central reading.^59^ It comprises four IUS parameters [Table 1] with near perfect interrater agreement. The score correlated with the global disease activity physician assessment.

These indices are promising, but external validation in a variety of large multicentre cohorts is needed before they can be adopted in clinical practice. To date, little prospective external validation has been undertaken [Table 2], and most studies that have attempted this have been hampered by their small sample size and retrospective nature with few highly specialised IUS operators.^60–62^ Dragoni *et al*. performed external validation of the IUS scores in a single-centre, prospective cohort of 73 patients using an endoscopic reference standard.^36^ The SUS-CD had a sensitivity of 93.3% and specificity of 71.4% for active CD, and the BUSS had sensitivity of 91.1% and and specificity of 82.1%. However, alternative cut-offs from the original descriptions were needed to achieve these performance characteristics. The IBUS-SAS had a sensitivity of 82.2% and specificity of 100% for detecting active CD and was statistically superior to the SUS-CD and the BUSS for identifying severe endoscopic CD. A limitation of this validation study was that all IUS was performed by a solitary experienced practitioner at a single centre. To address this, a recent study applied the SUS-CD and BUSS to patients from the prospective Magnetic Resonance Enterography or Ultrasound in Crohn’s disease [METRIC] trial; 111 patients had a histological reference standard and in 289 patients an MRE reference standard was used.^37^ The patients originated from eight different institutions, and IUS was performed and interpreted by one of 19 practitioners. Compared with histology, the sensitivity and specificity for active disease were 79% [69 to 86] and 50% [31 to 69] for SUS-CD, and 66% [56 to 75] and 68% [47 to 84] for BUSS, respectively. In comparison with the sMARIA, the sensitivity and specificity for active CD were 81% [74 to 86] and 75% [66 to 83] for SUS-CD, and 68% [61 to 74] and 85% [76 to 91] for BUSS, respectively. Given the diverse, multicentre, multireader study population, these findings are likely more generalisable estimates than others and approach expected performance in clinical practice. These activity scores need to be tested in further diverse populations, and treatment responsiveness in these settings also needs to be assessed.

## 4. The METRIC trial

The Magnetic Resonance Enterography or Ultrasound in Crohn’s disease [METRIC] trial is the largest, prospective, multicentre, cohort study to date that has provided a direct comparison of MRE with IUS.^11^ The trial, conducted across eight UK National Health Service [NHS] teaching and general hospitals, representative of routine clinical practice, compared the diagnostic accuracy of MRE and IUS for both the presence and the extent of active disease in newly diagnosed and relapsed CD. All patients underwent MRE and IUS, and a construct reference standard was used incorporating all relevant information obtained over a 6-month follow-up period [including clinical, biochemical, and endoscopic data]. This yielded an abundant and varied dataset. The key findings from the METRIC trial and related publications including secondary outcomes, and subsequent post hoc analyses that used the rich, multicentre, multireader data available from this pragmatic trial, are summarised in Table 3.^11,23,35,37,63,64,66^ One important outcome was the assessment of interobserver variability. Across the trial sites, 24 radiologists interpreted MRE and 19 performed IUS. One sonographer undertook IUS. All the radiologists had completed the Fellowship of the Royal College of Radiologists [FRCR], were affiliated to the British Society of Gastrointestinal and Abdominal Radiology [BSGAR], and had at least 1 year of subspecialty training in gastrointestinal radiology. The sonographer had received local formal training, was performing IUS routinely in their regular practice, and had 20 years of experience. The radiologists interpreting MRE had a median of 10 [interquartile range 6 to 11] years of experience, and practitioners interpreting ultrasound had a median of 8 [4 to 11] years of experience. During the trial, a median of 30 [20 to 45] MRE examinations and a median of 25 [12 to 40] IUS studies were undertaken at each trial site. Within the trial, there was reasonable agreement between radiologists for identifying small bowel disease presence on MRE for both newly diagnosed and suspected relapse cases, although agreement for disease extent was lower.^23^ IUS also showed substantial practitioner agreement for identifying small bowel CD in both newly diagnosed and suspected relapse patients.^63^

**Table 3:** Selected findings and insights provided by the METRIC trial dataset.

Reference	Objective	Number of participants	Study details	Results	Interpretation
Taylor *et al.*,^11^	To compare the diagnostic accuracy of MRE and US for SB CD	284 [133 ND, 151 SR]	Prospective multicentre cohort study	Sensitivity/specificity for SB disease presence: MRE = 97% [91–99]/96% [86–99] IUS = 92% [84–96]/84% [65–94] Sensitivity/specificity for SB disease extent: MRE = 80% [72–86]/95% [85–98] IUS = 70% [62–78]/81% [64–91]	Both MRE and US have high sensitivity for detecting SB disease presence
Bhatnagar *et al*.,^23^	To assess the interobserver variability for diagnosis of disease presence and extent of small bowel and colonic CD using MRE	73 consecutive patients [28 ND, 45 SR]	MRE read independently by three radiologists	Agreement for small bowel disease presence for ND/SR: 68% [κ = 0.36]/78% [κ = 0.56] Agreement for colonic disease presence for ND/SR: 43% [κ = 0.14]/53% [κ = 0.07] Agreement for colonic disease for ND/SR: presence was 61% [κ = 0.21 fair agreement] for ND/ 60% [κ = 0.20, slight agreement] for SR	There is a reasonable agreement between radiologists for small bowel disease presence using MRE for newly diagnosed Crohn’s disease, and patients with suspected relapse, respectively. Agreement is lower for disease extent
Bhatnagar *et al.*,^63^	To assess inter-observer variability for detection, extent and descriptive features of small bowel and colonic CD on IUS	38 [11 ND, 26 SR]	IUS performed by six practitioners	Agreement for small bowel disease presence for ND/SR: 82% [52–95], *κ = *0.64/ 81%, *κ* 0.63 Agreement for colonic disease presence for ND/SR: 64%, *κ* 0.27/ 78%, *κ* 0.56 Simple agreement between practitioners for disease presence: SB = 84% Colonic = 87%	There is substantial practitioner agreement for SB-CD presence in ND and SR patients
Bhatnagar *et al*.,^64^	To compare the distention quality and patient experience of oral mannitol and PEG for MRE	105	Overall and segmental bowel distention assessed by 2 independent radiologists	Per patient distension quality rated as ‘excellent’ or ‘good’: Mannitol = 54% [37/68] PEG = 46% [17/37] Jejunal distension rated as ‘excellent’ or ‘good’: Mannitol = 40% [27/68] PEG = 14% [5/37] Symptom tolerability was comparable between agents	Mannitol-based solutions and PEG achieve comparable distension quality and side effect profiles. Jejunal distension is better quality with mannitol. Neither distension quality nor side-effect profile is altered by ingestion of more than 1 L of mannitol
Kumar *et al*.,^35^	To compare the sMARIA, London, and ‘extended’ London indices for quantifying terminal ileal CD activity using a histopathological reference standard	111 [75 ND, 36 SR]	MRE activity indices were retrospectively derived	Sensitivity/specificity for active disease: sMARIA = 83%/ 41% London = 76%/ 64% ‘Extended’ London = 81%/ 41% Sensitivity/specificity of sMARIA for severe disease: 84%/53%	Compared with a histological reference standard, all three indices were sensitive for active TI CD [sMARIA for severe disease], but specificity was lower
Kumar *et al.*,^37^	To compare SUS-CD and BUSS against histological and MRE reference standards	111 [75 ND, 36 SR] for the histology reference standard 284 [133 ND, 151 SR] for the MRE reference standard	IUS activity indices were retrospectively derived	Against histology, sensitivity/specificity: SUS-CD = 79%/ 50% BUSS = 66%/ 68% Against MRE, sensitivity/specificity: SUS-CD = 81%/ 75% BUSS = 68%/ 85%	Particularly when compared with MRE activity scoring, SUS-CD and BUSS are promising tools in a real-world clinical setting
Miles *et al*.,^65^	To compare patient acceptability and burden of MRE and US with each other, and to colonoscopy	159	Patients completed an experience questionnaire on the burden of the investigations	Rated as very or fairly acceptable: MRE = 88% IUS = 99% Colonoscopy = 60% Recovery time: MRE longer than IUS, but shorter than colonoscopy Willingness to undergo repeat study: Patients were less willing to undergo MRE again than US, but more willing than for colonoscopy	MRE and US are well tolerated. Although MRE generates greater burden, longer recovery, and is less preferred than US, it is more acceptable than colonoscopy
Taylor *et al*.,^66^	To prospectively compare the diagnostic accuracy of SICUS and conventional US for SBCD extent	64	Patients had SICUS performed by the same practitioner who performed their conventional US	SB disease extent sensitivity/specificity: IUS and SICUS = 71%/ 86% Colonic disease extent sensitivity/specificity: IUS = 13%/ 82% SICUS = 17%/ 92%	SICUS does not improve the accuracy for SB or colonic disease compared with IUS

BUSS, Bowel Ultrasound Score; CD, Crohn’s disease; IUS, intestinal ultrasound; MRE, magnetic resonance enterography; ND, newly diagnosed; PEG, polyethylene glycol; SB, small bowel; SICUS, small intestine contrast enhanced ultrasonography; sMARIA, Simplified Magnetic Resonance Index Of Activity; SR, suspected relapse; TI, terminal ileum.

## 5. Developments and Controversies

### 5.1. Routine use of diffusion-weighted imaging in MRE

Diffusion-weighted imaging [DWI] is usually abnormal in bowel affected by IBD, reflecting the histopathological processes of inflammation, fibrosis, oedema, and vasculopathy due to the reduced molecular motion of water.^67^ This causes high signal on high b-value images, with corresponding low signal on the apparent diffusion coefficient [ADC] map. DWI is useful for detecting active inflammatory disease, but it cannot be used exclusively as fibrosis also causes restricted diffusion.^68,69^ Studies have demonstrated that whereas subjective assessment of DWI is very useful to highlight areas of abnormality that deserve close scrutiny on the other available sequences, it is not a robust method in isolation to define inflammatory CD.^70^ Furthermore, ADC values have poor intra- and interobserver variability.^71–73^ Reflective of that, recent data suggest that ADC values are insufficient when used alone to evaluate treatment responsiveness.^74^ Streamlining of the MRE protocol to reduce scan time, associated cost, and patient burden, while retaining high sensitivity and specificity, is a key priority; DWI is likely to face increased scrutiny and is currently considered an optional sequence [Box 1].^75,76^

**Box 1. Unanswered clinical and research priorities in the imaging of Crohn’s disease**

To establish which individual MRE and IUS parameters are most useful for assessing disease activity in routine clinical practice to optimise time-effectiveness

More dedicated multicentre, multireader studies to validate currently available MRE and IUS activity scores for both initial diagnosis and monitoring treatment responsiveness

Studies to better understand how to optimise MRE and IUS within clinical pathways to maximise diagnostic performance and influence clinical decision making, while maintaining cost-effectiveness and taking into account patient preference

Greater focusing of MRE protocols, thereby reducing the scan time

Reduce the subjectivity of MRE and IUS interpretation

Optimise training pathways to permit radiologists and gastroenterologists sufficient expertise in performing IUS/POCUS

Studies to establish whether MRE and IUS has a role in prognostication, for example whether baseline imaging can predict those who will develop severe disease

Translation of radiomics from the research setting to clinical practice

Development of parameters to allow quantification of small bowel motility on IUS

To establish how other applications of artificial intelligence could improve the performance and efficiency of imaging eg, automated segmentation of diseased bowel

MRE, magnetic resonance enterography; IUS, intestinal ultrasound; POCUS, point-of-care ultrasound.

### 5.2. Gadolinium-enhanced MRE

The decision to perform gadolinium-enhanced imaging varies across different institutions, even though consensus guidelines still recommend its use.^76^ However, there is accumulating evidence that in most cases this can be dispensed with, thereby avoiding the risk of gadolinium deposition and associated potential risks while also reducing the duration and cost of the study.^77^ In a post hoc analysis of a prospective trial, Rimola *et al*. considered 46 CD patient, comparing the accuracy of the sMARIA calculated with and without contrast-enhanced sequences in determining the response to biologics.^49^ The sMARIA with and without contrast had sensitivity of 76% and 80%, and specificity of 95.2% and 95%, respectively. Seo and colleagues assessed whether MRE performed with DWI in the absence of gadolinium was non-inferior to gadolinium-enhanced MRE for small bowel CD; in a cohort of 50 patients, they reported no statistical difference in the sensitivity and specificity of identifying active CD.^78^ These findings have since been replicated.^32,66,79^ Performing gadolinium-enhanced imaging can probably be reserved for patients with penetrating disease.^25^

### 5.3. Oral and IV contrast for IUS

Ingesting oral contrast medium before performing transabdominal IUS distends bowel loops, which improves visualisation of the bowel wall, and increases the separation between adjacent bowel loops. The technique is known as small intestine contrast-enhanced ultrasonography [SICUS] and has been extensively studied, with promising results.^80–85^ The incremental benefit over conventional IUS remains uncertain, and it is undoubtedly more laborious, time-consuming, and less acceptable to patients, which explains why it is not yet widely adopted.^66,86^ Nevertheless, SICUS is a useful technique for problem-solving and is currently only practised in centres with specialist expertise in this technique.

Contrast-enhanced ultrasound [CEUS], whereby contrast is administered intravenously before performing US, is another technique which has received some attention.^87–91^ It provides the ability to assess quantitative parameters related to bowel wall vascularisation, but its clinical usefulness is yet to be determined. Some preliminary data suggest CEUS may help distinguish between inflammatory and fibrotic disease in certain clinical situations, but these findings need to be reproduced in large, prospective studies.^92^ Another limitation of CEUS as a tool for quantifying CD burden is its lack of reproducibility, due to the lack of standardisation around probe/scanner combination and acquisition parameters used.^93^ Currently, CEUS is only performed in a few centres and is unlikely to be widely adopted unless significant benefit is demonstrated, given that is more invasive and time-consuming than conventional IUS. However, it is useful for characterising penetrating complications, particularly for distinguishing between a drainable abscess, which demonstrates enhancement only in the wall, and an inflammatory mass, which exhibits intralesional enhancement.^94^

### 5.4. Quantified bowel motility measurement

Fluoroscopic techniques have long demonstrated altered motility in bowel segments that are affected by CD, but quantification was not possible. However, modern 1.5 and 3 Tesla MR scanners can now assess small bowel motility in a single breath-hold, and post-processing software permits quantification. Despite IUS offering real-time assessment of bowel motility, this is subjective as there are currently no reliable methods to quantify motility by US. The ability to quantify small bowel motility by MRE has generated significant interest in its clinical utility. Several studies have shown that a reduction in small bowel motility measured by MRE is correlated with histopathological and endoscopic activity, and the recovery of motility may be a useful marker for treatment response.^95–99^ Results from the MOTILITY trial [ISRCTN14481560] will determine how effective small bowel motility measurements are in predicting treatment response at 1 year in patients with small bowel CD, who are starting biologic treatment.

### 5.5. Predictive potential of cross-sectional imaging

The ability to accurately identify at initial diagnosis CD patients who are most at risk of developing future severe CD complications [including stricturing and penetrating disease, and risk of intestinal surgery] represents a major unmet clinical need. The development of a robust predictive tool would allow prioritisation for early advanced medical therapy [Box 1].^100^ Although clinical predictors for the development of severe disease have been identified, these lack specificity, and to date, prognostic research evaluating cross-sectional imaging is lacking.^101^

Fiorino and colleagues assessed the prognostic role of MRE in CD patients who were within 2 years of their initial diagnosis.^102^ They found that bowel damage [presence of stricture, fistula, or abscess] on imaging was associated with progression to surgery and more frequent future hospitalisation. Similar results were reported in a cohort of 112 CD patients who had established disease, suggesting they were not necessarily imaged at the time of diagnosis.^103^ In a single-centre study of 52 CD patients at any time in their disease course, findings on outpatient MRE of either restricted diffusion, increased upstream dilatation from a stricture, complex fistula, peri-enteric inflammation or fibro-fatty proliferation, and increased length of disease involvement, were associated with progression to surgery.^104^ These results are not surprising, as MRE is uniquely placed to assess both bowel damage and inflammation simultaneously, unlike common biomarkers such as C-reactive protein [CRP] and faecal calprotectin. To date, no study has assessed whether baseline MRE at initial diagnosis can predict disease trajectory, but this will soon be rectified.^105^

IUS is also a candidate for predicting CD trajectory at the time of diagnosis. Bowel wall thickness >7 mm predicts progression to surgery within 1 year.^106^ As with MRE, the presence of a stricture, fistula, or abscess on IUS at any time in the disease course is associated with progression to surgery within 12 months.^56^ More work is needed to see if baseline IUS at the time of diagnosis has prognostic potential.

### 5.6. Point-of-care ultrasound

Point-of-care ultrasound [POCUS], which refers to diagnostic ultrasonography performed at the bedside, is well established in a few specialties such as rheumatology in the outpatient setting. There is increasing interest in its adoption within gastroenterology clinics for the assessment and monitoring of CD and UC.^107,108^ Studies assessing the accuracy of POCUS in IBD have demonstrated a sensitivity for detecting active disease ranging from 87.5% to 91% and specificity of 61.1% to 91.9%, compared with MRE and colonoscopy reference standards.^56,109–111^ These studies included operators with a wide range of experience in IUS, from those who had performed 200 scans to experts with experience in performing several thousand studies. The use of POCUS does influence decision making, with 58–60% of patients with CD having a change in their management plan made because of the examination.^112^ Furthermore, around half of asymptomatic patients were found to have active disease on POCUS. In a retrospective review of a specialist centre’s experience of POCUS for 345 examinations, 60% of these led to a change in clinical decision, with almost 50% resulting in a treatment change.^113^ Correlation with MRE or colonoscopy was 80–86.3%, with no moderate or severe disease missed. A study in Canada showed that an individual could deliver POCUS with adequate sensitivity and specificity compared with MRE after completing 200 supervised scans in a high-volume IUS centre.^111^

**Figure 2:**
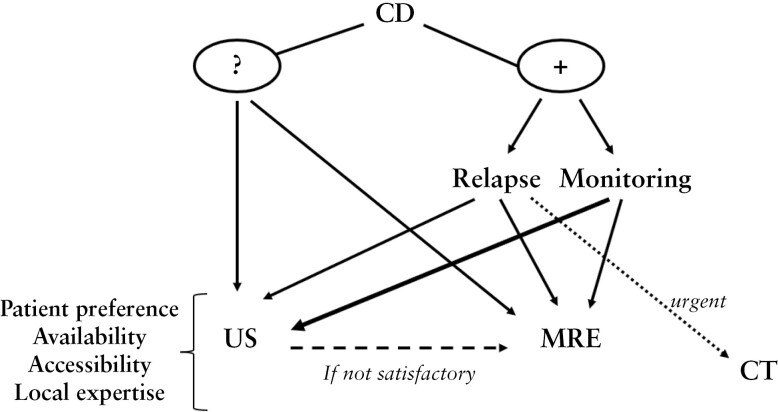
Proposed diagnostic algorithm. - Suspicion of CD: mainly IUS - Known CD: - Active relapse: IUS/MRE depending on disease phenotype; CT in acute setting - Asymptomatic patients for monitoring: mostly IUS At any point: additional MRE if IUS is not satisfactory. CD, Crohn’s disease; IUS, intestinal ultrasound; MRE, magnetic resonance enterography; CT, computed tomography.

Large prospective studies are needed to confirm the robustness of POCUS, and clearly defined standards of training are essential. In 2016, the World Federation for Ultrasound in Medicine and Biology [WFUMB] published a position paper calling for the formulation of a curriculum and establishment of minimum core competencies for IUS training. These courses are being introduced worldwide.^114^ A consensus statement on competency criteria required to be able to deliver IUS has recently been published.^115^ POCUS is likely to be most effective for regular follow-up and treatment monitoring in simple CD. However, in the case of complex phenotypes such as penetrating, fistulising, and stricturing disease, MRE should be preferred [Figure 2].

### 5.7. Handheld ultrasound devices

Handheld ultrasound [HHUS] devices have been investigated for IBD. HHUS was compared with IUS, showing promising sensitivity of 92% for thickened bowel wall and 94% for length of disease.^116^ Reproducibility between two separate clinicians was similar to conventional IUS [Cohen’s kappa coefficient 0.84–0.85]. HHUS has been compared with MRE for new patients referred to a tertiary IBD unit with high suspicion of CD, with a diagnostic sensitivity of 87.5% compared with 91.67% by MRE, with no statistically significant difference.^109^ MRE was superior to HHUS for extent, location, and complications. These studies suggest that handheld devices could be used as a screening tool for patients at risk of IBD, and as a monitoring tool for disease activity, although the images are less clear than those obtained using portable or departmental US machines.

### 5.8. Cost

IUS is inexpensive, quicker to complete than MRE, and generates a result at the time of the test. Although it is expected that the adoption of IUS would bring about substantial cost savings by reducing MRE and endoscopy usage, there are limited data to back up real-world cost savings of IUS use and POCUS. A centre with limited IUS availability estimated an almost £500 000 saving if IUS was used as an alternative to MRE or ileo-colonoscopy for patients suitable for the test, with minimal missed pathology.^117^ However, in a METRIC trial sub-study, Taylor *et al*. reported no significant differences in cost, outcomes, and net monetary benefit overall between the two options in both newly diagnosed patients and those with suspected clinical relapse.^66^

Data regarding the cost-benefit of incorporating POCUS into the clinic are required to determine if it reduces outpatient investigations and the number of clinical appointments. The potential cost savings must be compared with the capital cost of acquiring the machine and the time required for clinician training, as well as the increased time taken to perform POCUS during a clinic appointment.

### 5.9. Artificial intelligence

The present interpretation of cross-sectional imaging relies upon subjective assessment by radiologists and is thus at risk of interobserver variability. Advances in technology may permit automated or at least semi-automated intestinal segmentation that should reduce variability [Box 1].^118^ This, in turn, may result in the automated extraction of standardised, clinically relevant parameters that assess CD activity.^119–121^ However, this is likely to be challenging, exemplified by two metanalyses that demonstrate that artificial intelligence [AI]-based solutions are far less often introduced to abdominal imaging compared with other imaging subspecialties.^122,123^ Nevertheless, there are emerging data that show promise. In a cohort of 121 patients, Ding *et al.* found a radiomics model to be objective and reproducible, and comparable to the MARIA performed by a senior radiologist.^124^ Liu *et al.* developed a machine learning method for predicting ileal CD through radiomic features of bowel wall and mesenteric fat from T2-weighted MRE, and compared its performance with expert radiologists.^125^ In their cohort of 135 patients, radiomic features could identify the presence of CD with 89.6% accuracy, compared with an accuracy of 83.7–88.1% of three expert radiologists with up to 14 years’ experience. In a pilot study, Chirra *et al.* identified radiomic features from MRE that accurately stratified patients into high-risk and low-risk groups, based on the need for surgery within 1 year of imaging.^126^ Combining radiomic features with clinical variables and the sMARIA produced a highly accurate multivariate prognostic model for predicting time to surgery. Translation of radiomics beyond the research setting and into clinical practice remains an ongoing challenge and future priority [Box 1].^127^ Carter and colleagues showed that deep learning, using a convolutional neural network, can accurately identify US signs of IBD activity.^128^ Such technology may help more inexperienced operators, with the potential to ultimately permit automated detection of bowel inflammation and greater standardisation of US imaging interpretation. External validation in independent cohorts is the next step.

## 6. Selecting between MRE and IUS

The decision regarding which cross-sectional technique to employ is multifaceted and depends on patient characteristics, the clinical question, scanner and interpretative expertise availability, and patient preference.^6,129^ In general, all tests have their strengths and limitations, and the question is not binary but rather which test is most suitable for a particular patient at a particular point in their disease course.^129^ Essentially, MRE and IUS are complementary in clinical practice.

As with all imaging investigations, high-volume sites develop expertise in a particular test, which is an important consideration as all tests have an interpretative learning curve. In general, CT, due to its use of ionising radiation, should be avoided outside the acute setting, especially for repeat/follow-up investigations.^2,15,130,131^ Meta-analysis suggests IUS and MRE are broadly similar in terms of diagnostic accuracy,^21,132^ although prospective multicentre head-to-head comparison suggests MRE has greater accuracy, particularly for staging the location of small bowel CD, and is perhaps preferred at the time of diagnosis when the disease distribution and phenotype are first defined.^11^ IUS, however, tends to perform better in the colon. Both MRE and IUS have proven utility in disease follow-up and assessing treatment response, and the simplicity, patient acceptability and immediacy of IUS, particularly at point of care, makes it an attractive option if available, particularly in established, non-complex disease phenotypes. IUS and MRE are also both highly effective for identifying intra-abdominal complications in CD.^133,134^

An important consideration when selecting the most appropriate imaging investigation is patient experience and preference. In the METRIC trial, the burden of MRE, albeit low, was significantly greater than IUS.^65^ Recovery times for MRE were longer and patient willingness to undergo the test again also lower for MRE [91% vs 99% for IUS]. Nevertheless, MRE was consistently rated as preferable to colonoscopy, and patients rated diagnostic accuracy as the most important test attribute. Similarly in an Australian study, IUS was considered to be highly acceptable, well tolerated by patients, and their preferred tool for monitoring CD.^135–137^

We provide a potential algorithm for integrating MRE and IUS into routine clinical practice in Figure 2. For patients who are suspected of having a new diagnosis of CD, IUS is often preferred as a ‘screening’ tool, although MRE remains an appropriate choice too. For patients with known CD, MRE is generally favoured for the diagnosis of relapse, particularly in complex disease phenotypes, although IUS can also be used. Where there is concern for an acute abnormality, CT should be considered, particularly if this facilitates rapid diagnosis. For regular monitoring, during a course of therapy for example, IUS is very well suited. In all instances, if the images from the IUS study are unsatisfactory, for instance due to body habitus or obscuration from bowel gas, MRE should be performed.

## 7. Conclusions

There is overwhelming evidence that supports the role of cross-sectional imaging in diagnosing, monitoring, and assessing treatment response in CD. These non-invasive, radiation-free techniques are tolerated well by patients and highly sensitive and specific, and their use is constantly evolving. External validation of activity scores in independent cohorts will help standardise reporting and increase objectivity and reproducibility. Coupled with the plethora of technological advances, MRE and IUS are likely to contribute significantly to improved patient outcomes and the delivery of more personalised treatment in CD. A collaborative multispecialty approach, with routine integrated clinics and close communication between the treating gastroenterologists and radiologists regarding all aspects of the patient’s imaging and management plan, would be an effective means of achieving this outcome.

## Supplementary Data

Supplementary data are available at *ECCO-JCC* online.

jjae042_suppl_Supplementary_Table_S1

## Data Availability

No new data were generated or analysed.
