# Supplementary material for: Magnetic Resonance Enterography and Intestinal Ultrasound for the Assessment and Monitoring of Crohn’s Disease
Source: J Crohns Colitis. 2024 Mar 30;18(9):1450–63. doi: 10.1093/ecco-jcc/jjae042 (PMC11369078; doi:10.1093/ecco-jcc/jjae042)
Supplement: jjae042_suppl_Supplementary_Table_S1 [file jjae042_suppl_supplementary_table_s1.docx]

| **IUS** | **MRE** |
| --- | --- |
| **Initial systematic scan** | **Minimum sequences** |
| • Convex probe (low frequency 3,5-5 MHz) | • Coronal SSFPGE without FS  • Axial and coronal T2 FSE without FS  • Axial or coronal T2 FSE with FS |
| **Detailed visualisation of intestinal wall** | **Optional sequences (add 2 of 3)** |
| • Linear probe (high frequency 6-11 MHz)  • Colour Doppler US to assess vascularisation of pathological segments, low flow range (5-7 cm/s) | • Axial and coronal pre- and post- contrast 3D T1-weighted GE with FS (60-70 s)  • Axial DWI (b-values 50 and 600)  • Coronal Cine Balanced SSFP |

**Supplementary Table 1. IUS standard examination protocol and MRE imaging protocol for both 1.5 and 3 Tesla scanners.**

SSFPGE= steady-state free precession gradient echo; FSE= fast spin echo; FS= fat saturation; 3D= 3-dimensional; GE= gradient echo; DWI= diffusion-weighted imaging; SSFP= steady-state free precession
